# Antibiotic Stress, Genetic Response and Altered Permeability of *E. coli*

**DOI:** 10.1371/journal.pone.0000365

**Published:** 2007-04-11

**Authors:** Miguel Viveiros, Myrielle Dupont, Liliana Rodrigues, Isabel Couto, Anne Davin-Regli, Marta Martins, Jean-Marie Pagès, Leonard Amaral

**Affiliations:** 1 Unit of Mycobacteriology, Unidade de Parasitologia e Microbiologia Médicas (UPMM), Instituto de Higiene e Medicina Tropical, Universidade Nova de Lisboa, Lisboa, Portugal; 2 UMR-MD-1, IFR48, Facultés de Médecine et de Pharmacie, Université de la Méditerranée, Marseille, France; 3 Centro de Recursos Microbiológicos (CREM), Faculdade de Ciências e Tecnologia, Universidade Nova de Lisboa, Lisboa, Portugal; The Research Institute for Children, United States of America

## Abstract

**Background:**

Membrane permeability is the first step involved in resistance of bacteria to an antibiotic. The number and activity of efflux pumps and outer membrane proteins that constitute porins play major roles in the definition of intrinsic resistance in Gram-negative bacteria that is altered under antibiotic exposure.

**Methodology/Principal Findings:**

Here we describe the genetic regulation of porins and efflux pumps of *Escherichia coli* during prolonged exposure to increasing concentrations of tetracycline and demonstrate, with the aid of quantitative real-time reverse transcriptase-polymerase chain reaction methodology and western blot detection, the sequence order of genetic expression of regulatory genes, their relationship to each other, and the ensuing increased activity of genes that code for transporter proteins of efflux pumps and down-regulation of porin expression.

**Conclusions/Significance:**

This study demonstrates that, in addition to the transcriptional regulation of genes coding for membrane proteins, the post-translational regulation of proteins involved in the permeability of Gram-negative bacteria also plays a major role in the physiological adaptation to antibiotic exposure. A model is presented that summarizes events during the physiological adaptation of *E. coli* to tetracycline exposure.

## Introduction

Intrinsic antibiotic resistance in Gram-negative bacteria (without chromosomal mutation or the acquisition of mobile genetic elements encoding resistance determinants) can be increased by preventing the antibiotic from entering the cell. This can be achieved by the control of the outer membrane permeability and by the effectiveness of the efflux (active pumping out) of antibiotics [1]–[3]. The effectiveness of the outer membrane of Gram-negative bacteria as a barrier only delays the influx of various antibiotics, detergents and dyes. Intrinsic resistance to antibiotic agents is brought about by efflux pumps, which extrude the drug from the periplasmic space to the environment, enabling bacterium to survive in the presence of these noxious agents [1], [4]. Additional resistance is afforded by over-expressed efflux pumps that extrude a wide variety of unrelated antibiotics. Over-expressed efflux pumps of Gram-negative bacteria result in a multidrug resistant (MDR) phenotype known to be a prevalent form of clinical resistance [3].

We have previously demonstrated that it is possible to induce high-level resistance to tetracycline (TET) in susceptible *Escherichia coli* K-12 by a gradual, step-wise increase in the exposure to the antibiotic [5]. The induction process takes about 110 days and this resistance can be reversed by either transfer to drug free medium or by the use of Phe-Arg-napthylamide (PAβN), an inhibitor of the AcrAB efflux pump system [3]–[5]. The nine major inner membrane transporter genes of *E. coli* K-12 were over-expressed after prolonged exposure to TET, with the *acrB* being the most expressed transporter gene and a clear connection between the induced activity of the AcrAB system and TET induced resistance was demonstrated [5]. Besides becoming resistant to TET, the induced strain became resistant to a variety of other antibiotics, detergents and dyes that are not substrates of the AcrAB system [3]–[5]. The development of this MDR phenotype led us to explore and analyse the interplay between the major efflux pump systems present in *E. coli* and the control of the outer membrane permeability through the regulation of the porin channels.

In *E. coli*, outer membrane permeability is regulated by the balance of porin proteins, the diffusion channels that are the major route for passage of small hydrophilic compounds [1], [6], [7]. The two major outer membrane proteins (OMPs) in *E. coli* are OmpC and OmpF, consisting of three 16-stranded β-barrels defining a transmembrane pore in the outer membrane porin [8], [9].

Highly expressed under optimal environmental conditions, their level of expression is adjusted when it is necessary to minimize penetration of noxious compounds or maximize access to nutrients [7], [10], [11]. It has been demonstrated that the level of expression of the porins OmpC and OmpF not only controls the permeability of the outer membrane to glucose and nitrogen uptake under nutrient limitation [10], [11], but may also be differentially regulated by the concentration of certain antibiotics in the environment [4], [12], [13]. The OmpC and OmpF coding genes are transcriptionally regulated by a two-component signal transduction regulatory system consisting of the OmpR and EnvZ proteins [14]. Recently, it has been shown that the over-expression of OmpX, structurally related to the eight-β strand OmpA (a major OMP involved in the stabilization of the bacterial membrane), leads to a decrease in the expression of OmpC and OmpF porins and a decreased susceptibility to beta-lactams and other antibiotics in *E. coli* [15]. Because mutants with decreased expression of porins show only small increases in the minimum inhibitory concentration (MIC) of relevant antibiotics, the complete shut down of influx of small molecules into *E. coli* does not readily occur [16].

*E. coli* has been shown to have at least nine distinct proton-dependent efflux pump systems that bestow resistance to two or more antibiotics (MDR). The genes coding for each of these efflux pumps are *emrE* [17], *acrEF* (formerly *envCD*) [18], *emrAB* [19], *emrD* [20], *acrAB-tolC* [21], *mdfABC* [22], *tehA* [23], *acrD* (an *acrB* homolog) [24] and *yhiUV* [25]. They belong to one of three genetically and structurally defined families: the major facilitator superfamily (MFS – *emrD, mdfA, emrB*), the resistance nodulation-cell division family (RND- *acrB, acrF, acrD, yhiV*), and the small multidrug resistance family (SMR – *emrE, tehA*) [3], [4]. The tripartite AcrAB-TolC system is the most well-studied MDR pump system consisting of an inner membrane efflux transporter (AcrB) that removes antibiotics from the cytoplasm to the periplasm, where the linker protein (AcrA) directs the inter-membrane transport of the antibiotic through the outer membrane channel (TolC) to the environment [1], [3], [6]. The major efflux pump systems in *E. coli* are from the RND family and have broad substrate specificity. Their expression is controlled by systemic transcriptional activators like the MarA, encoded by the multiple antibiotic resistance operon *marRAB* [26], and homologs like SoxS and Rob [4], [27]. MarA not only controls the expression of the efflux pump systems, but is also involved in the control of porin expression (by decreasing it) through the activation of *micF*, a small antisense RNA that binds with *ompF* mRNA preventing its translation, and activates the expression of the porin expression down-regulator OmpX [15], [28]. These global activators, when induced by oxidative stress or the presence of noxious compounds in the environment, enhance resistance of enterobactereaceae to a variety of antibiotics, hence an MDR phenotype [4], [31], [37]. Moreover, they control the degree of intrinsic resistance of enterobactereaceae and increase the level of efflux pump expression. The regulation of porin level and expression of MDR efflux pumps has been suggested to occur by a common pathway and/or a cascade of events [4], [29].

Our studies of step-wise induction of TET resistance, by gradual exposure of *E. coli* K-12 wild-type to TET, may afford an understanding of the genetic regulation of MDR efflux pumps, their interplay, and relationship to the permeability barrier, all of which are involved during the TET resistance induction process [5]. Therefore, with the aid of quantitative real-time reverse transcriptase-polymerase chain reaction (RT-PCR) methodology and western blot detection we have analysed and correlated the activity of regulatory genes that affect the MDR phenotype, of genes that code for transporter proteins of RND efflux pumps, of genes that code OMPs; and the level of OMPs during the process of induced resistance of *E. coli* K-12 wild-type by prolonged exposure to increasing concentrations of TET.

## Results and Discussion

**Figure 1:**
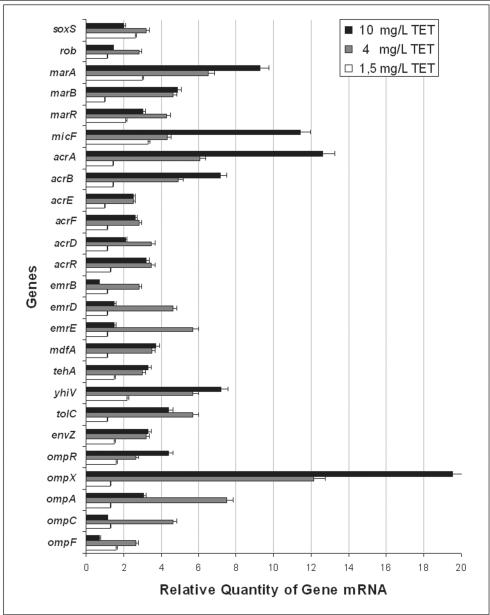
Relative expression of outer membrane proteins, regulators and inner membrane transporter genes. Data from three independent total mRNA extractions of *E. coli* AG100 physiologically adapted to increasing concentrations of TET compared to its parental non-induced strain grown in the absence of TET as described in Materials and Methods. A ratio of 1 corresponds to no alterations in expression compared with untreated control cells. Values were corrected for standard deviation range.

The results obtained in this study and presented by Figure 1 are discussed in terms of relationships that have been established for regulatory and responding genes.

### Genes responding to stress:

#### soxS and rob

After resistance to TET has been established at the highest initial concentration of the antibiotic (*i.e.* 1.5 mg/L), the response of the *soxS* gene is 2.8 times more active than that of the unexposed control. This response is further increased to 3.5 times after resistance to 4 mg/L has been induced. However, by the time the strain has become resistant to 10 mg/L of TET, the response of the gene has been reduced to a level below that initially observed, suggesting that the stress gene *soxS* performs its functions quite early under conditions of antibiotic pressure. The activity of the *rob* gene during the process of TET induced resistance is significantly increased after the bacterium becomes resistant to 4 mg/L of TET. As was the case of the other stress-response gene *soxS*, the increased activity noted is apparently not required for higher levels of resistance (*i.e.* 10 mg/L). Although *rob* has been reported to respond to exposure to solvents, detergents and metals [30], [31], in the current study an antibiotic response is included. The parallel response of both stress-responding genes noted in our study supports the conclusions of Michan *et al.* [32].

#### marA, marB and marR

The regulatory product of gene *marR* is known to down-regulate the activity of genes *marA* and *marB* by binding to the promoter region of the operator *marO*. Because TET is known to bind to the product of *marR*, and this produces an MDR phenotype [33], once the repressor activity is inhibited, the universal regulator *marA* would be expected to increase its activity. This expectation is confirmed by data in Figure 1. In this case, one can see that of all of the regulator genes, it is *marA* which is increased to the highest level (9.7 fold) at the time that the organism has developed resistance to 10 mg/L of TET.

Although nothing is known of the role of *marB* during MDR phenotypic expression, our study suggests that its function may precede that of *marA*. At this time, other than noting an increased activity of both of these genes, we do not know their precise relationship during the development of TET resistance that results from prolonged exposure to increasing concentrations of this antibiotic.

#### micF

Function of *micF* has been attributed to down-regulate OMPs [34] and is activated by *marA, rob* and *sox* [35]. The increase of activity of *micF* reaches its maximum level when the organism has become resistant to 10 mg/L of TET and parallels the rise of activity of *marA*. This behaviour of *micF* is consistent with that illustrated by others [28]. The over-production of MicF has been previously reported to decrease the amount of *ompF* mRNA [35]. We noted a three fold decrease of porin mRNA after resistance to 4 and 10 mg/L TET had been induced (Figure 1). This variation may be caused by the MicF effect on *ompF* mRNA; similarly MicC may have the same effect on *ompC* mRNA stability. In contrast, it is important to note that the level of *ompF* mRNA and *ompC* mRNA of TET exposed cells remained, at least, similar to that observed for the untreated control (ratio of 1 at 10 mg/L of TET).

### Efflux pump genes

#### *acrAB* and the other efflux pump transporter genes

The response of the *acrAB-tolC* when the organism is initially exposed to increasing concentrations of TET below that of its MIC of 2 mg/L, such as 1.5 mg/L, is marginal. This suggests that the activity of this operon under initial conditions of stress imposed by the exposure to low levels of TET is sufficient for the organism to escape from harm. With further exposure to increasing concentrations of TET, the expression of *acrAB-tolC* increases as the organism becomes more and more resistant to the antibiotic. The response of the other efflux pump transporter genes is one of increased activity. However, with the exception of the *yhiV* efflux transporter gene, an RND type efflux pump in *E. coli* with significant homology to AcrB [36], the level of activity expressed by the other efflux systems is much less than that evident for the *acrB* and *yhiV*. In addition, the other efflux pump genes express the highest level of activity when the organism has reached a level of resistance of 4 mg/L and with further increase of resistance, their level decreases. This suggests that the stress imposed during the early stages of exposure to TET requires the cooperation of all of the efflux pumps and that, as the level of activity of the two main efflux pumps of the bacterium is increased, there is a reduction in activity of the other pumps. The increased activity of *acrAB-tolC* and the increased synthesis of AcrA detected by immunoblot in TET induced cells (data not shown), parallel the increased activity of the regulator *marA*; a relationship that is consistent with that proposed by Barbosa *et al* [28].

### Stress regulator genes of OMP

The *ompR* and *envZ* genes are regulators of OMPs that permit hydrophilic compounds to readily enter the cell. *ompR* and *envZ* belong to the two-component signalling family and modulate gene activities of *ompF* and *ompC*, the two major OMP genes that code for the tri-barrel porin [7]. When *E. coli* is placed under stress, a cascade of gene activities is initiated, involving several global regulators such as MarA and MicF, which result in the down-regulation of porins [11], [27], [34]. This down-regulation results in decreased activity of *ompF* and *ompC*. As shown in Figure 1, whereas the increase in the expression of the *ompR* and *envZ* genes is maintained for the duration of exposure to increasing concentrations of TET, the response of the *ompA, C* and *F* genes is transiently increased and subsequently reduced to levels comparable to those of the *E. coli* cells that were not exposed to TET.

Because *micF* is considered to be a post-transcriptional regulator of porins, the activity of *ompF* and *ompC* may be related to the expression of this gene. Interestingly, we observed a high increase in the *micF* expression in TET induced cells (Figure 1). MicF binds the *ompF* mRNA generating an RNA duplex that alters the translation and mRNA stability. In the case of a MicF multi-copy producing strain, a putative factor (factor X) is believed to be essential for *ompF* mRNA destabilization and degradation [37]. Since over-production of MicF is observed in TET induced cells, a decrease of *ompF* mRNA would be expected as described recently with OmpC [38] (compare TET induced/control cell). In contrast, the porin mRNA level is similar to that produced in untreated cells. We may assume that as previously reported [37], the factor X becomes limited and cannot induce porin mRNA degradation.

**Figure 2:**
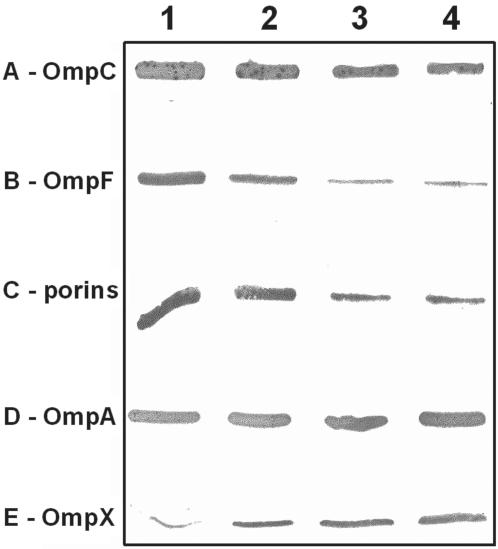
Immunodetection of the outer membrane proteins. The detections were carried out using antisera prepared against OmpC porin (A), OmpF porin (B), antigenic peptide located inside the internal loop 3 porin (C), OmpA (D) and OmpX (E) respectively. Immunodetection were carried out with total cell extracts from non-induced AG100 (1, 2) and 10 mg/mL TET-induced (3, 4) strains grown in LB and MH media (odd and even lanes respectively).

The analyses of genes involved in the increased resistance to TET suggest that the up-regulation of efflux pump genes is accompanied by a decrease of OmpF and OmpC synthesis. Evaluation of this suggestion was made by the use of immunoblot analyses of OmpC and OmpF proteins of the strain that is resistant to 10 mg/L of TET. As evident from Figure 2, OmpC is reduced and OmpF is markedly reduced in the strain that has become resistant to 10 mg/L of TET. The observed alterations in porin content were confirmed by the use of the antibody that recognizes the specific internal loop domain of general porins (Figure 2). These results are consistent with the notion that when the bacterium is placed under antibiotic stress, in conditions that permit it to adjust (namely slow exposure to sub-lethal concentrations of the antibiotic and nutrient availability), resistance is increased by the up-regulation of efflux pumps and down-regulation of porins.

#### OmpA and OmpX

OmpA is considered to be a structural OMP that contributes to the integrity of the cell envelope as a tri-barrel structure [39]. It does not appear to have a role in functions normally attributed to porins. In our study, there was a transient increase of *ompA* expression when the organism became resistant to 4 mg/L of TET (Figure 1). It may well be that as the exposure to increasing concentrations of TET reduces protein synthesis, the need for structural strengthening of the cell envelope takes place. However, with the increased effectiveness of efflux pumps and down-regulation of porins C and F, fewer molecules of antibiotic would be expected to reach their ribosomal targets even when resistance to TET has increased to 10 mg/L, and the extra need for OmpA is obviated.

**Figure 3:**
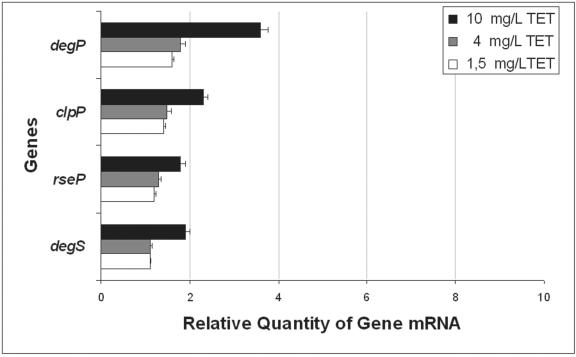
Relative quantification of the expression level of the protease genes. Data from three independent total mRNA extractions of *E. coli* AG100 physiologically adapted to increasing concentrations of TET compared to its parental non-induced strain grown in absence of TET as described in Materials and Methods. A ratio of 1 corresponds to no alterations in expression compared with untreated control cells. Values were corrected for standard deviation range.

The *ompX* gene codes for the outer membrane protein OmpX and over-production of this protein induces a reduction of the porin level in *Enterobacter aerogenes* [15]. As is evident from the results presented, the activity of *ompX* is the highest of all of the genes evaluated (Figure 1). Because the level of OmpX detected is also increased in TET induced strains (Figure 2), we propose that the regulatory role for this OMP involves a direct effect on porin assembly. Two hypotheses may be proposed: 1) OmpX alters the normal synthesis of OMPs, or 2) a component such as a chaperone is required for the construction of nascent porin [39]. Concerning the first hypothesis, no modification of OmpA synthesis was noted in TET induced strains (Figure 2) suggesting a more specific effect of OmpX on the porin expression. In this respect, the over-production of OmpX + TolC in the TET-10 mg/L strains may induce a saturation of OMP chaperones, such as YaeT and YfiO, that are necessary for the insertion of stably folded proteins into the outer membrane and subsequent construction of the tri-barrel porin [39], [40]. The increase of OmpX may then impair the normal assembly of porins. The unstable unfolded porin monomers will then be degraded by Deg proteases, serine-type proteases that play an important role in the proteolysis of misfolded and damaged proteins, to avoid toxic accumulation of abortive membrane protein [13], leading to drastic decrease of porin content as has been observed in the TET induced cells. This hypothesis is supported by recent data showing competition between TolC and porins during assembly [41] and by the role of DegP protease that removes the mis-folded membrane proteins accumulated within the periplasm [13], [41]. In addition, the degradation of mis-assembled unfolded forms of porin occurs very rapidly due to their unstable conformation [42]. Interestingly, an increased activity of genes that code for proteases in *E. coli* [41], [43] was noted in our study (Figure 3). The activity of *degP, clpP, rseP* and *degS* is increased from two to four fold after the organism has become resistant to 10 mg/L of TET and may account for the large reduction of porins due to the degradation of unfolded forms of OmpC and OmpF (Figure 2).

Living organisms have the capacity to adapt to changing environments without the need to rely on mutations, which are infrequent and thereby slow, to be incorporated into a population in a given environment. In the case of the efflux of toxic compounds, physiological adaptation of a cell to a given substance in a given environment begins with an event that takes place at or within the cell envelope and results in a sensor type of stress response. This eventually results in genetic activity that encodes for additional units of that same efflux pump that extrude a broad range of substrates. The addition of more efflux pumps into the cell envelope increases the survival of the organism. This scenario can be mimicked in the laboratory by the gradual, step-wise increase in the concentration of an antibiotic that permit members of the population to sequentially respond by adding more and more pump units to the cell envelope. In this study, *E. coli* AG100 becomes increasingly resistant to TET when exposure to TET is gradually increased. The characterisation of these TET induced strains by our study shows that the increased expression of efflux pumps is not the only mechanism involved in the physiological adaptation processes to TET pressure. There is a well-regulated and coordinated interplay between events at the genetic level and protein folding that decrease permeability of the cell envelope and increase efflux pump activity.

**Figure 4:**
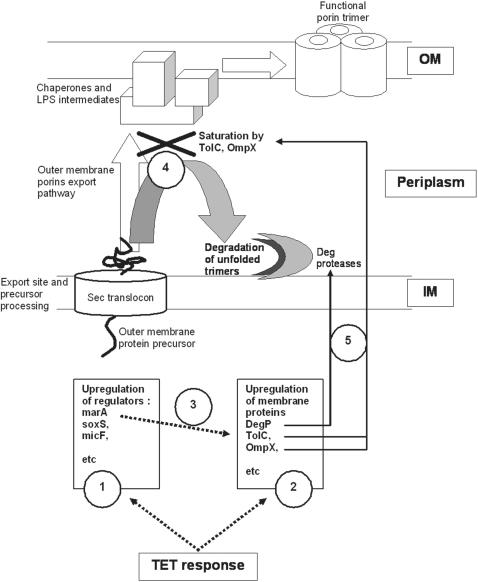
Tetracycline activation cascade of *E. coli* resistance physiological adaptation. Broken arrows indicate the activation in 1 and 2 over-expression of specific gene (direct TET pressure effect), in 3, the regulation by induced regulators (second level of control), in 4 the effect of activated genes coding for membrane proteins (third level of effect). Thick arrows (5) illustrate the effect of over-production of OMPs and proteases.

In the presence of initial non-lethal concentrations of TET, the wild-type *E. coli* reacts through the activation of early stress responses as seen by the immediate increase of the global regulators like MarA, SoxS, Rob and the activation of membrane and periplasmic proteases that release sigma factors in order to regulate the two major outer membrane proteins OmpC and OmpF. Following this initial stress response, a long-term adaptative response becomes noticeable with a sustainable increase of MarA that is not followed by the other two global regulators (SoxS and Rob) and, instead, is followed by two specific down-regulators of OmpC and OmpF expression, MicF and OmpX. Concomitantly, the over-expression of MarA leads to the transcriptional activation of AcrAB-TolC expression, the major efflux pump system of *E. coli* along with an increased expression of the other efflux systems. This is the basis for the development of an MDR phenotype [4], [44], [45]. The gradual step-wise physiological adaptation of *E. coli* to TET forces the cell to answer to a constant stressful environment by the activation of a cascade of long-term events that are summarized in Figure 4.

This is the first report that describes, in addition to the transcriptional regulation of genes coding for membrane proteins, a post-translational regulation of proteins involved in the membrane permeability in Gram-negative antibiotic resistant bacteria. This double control that severely reduces the amount of porins of the outer membrane is directly connected with the production of proteases which eliminates the non-assembled trimers of porins. Therefore, the reduced permeability of the TET induced resistant strains, in conjunction with the increased expression of the efflux pumps, guarantees not only the survival of these cells in the presence of TET, but also accounts for the MDR phenotype shown by these cells [5].

The physiological adaptation which results in an MDR phenotype should be taken into account when dealing with MDR *E. coli* infections, as these mechanisms of low-level resistance can be underestimated and ultimately result in high-level, clinically relevant resistance, not only in *E. coli* but also in other bacteria [45]. Because the process of MDR physiological adaptation is slow, the adjustment of the antibiotic dose to a level which exceeds the capacity of the bacterium to survive, without reaching levels that are toxic for the patient, may yield a positive outcome. This has been experienced by clinicians who claim cures with antibiotics for which resistance has been reported by the laboratory. Therefore, the quantification of efflux activity that renders the bacterium MDR [46] may provide relevant information for therapeutic guidance.

## Materials and Methods

### Materials

Tetracycline (TET) and Phe-Arg-napthylamide (PAβN) were purchased from Sigma Aldrich Quimica SA, Madrid, Spain. TET solutions were prepared in methanol whereas PAβN solutions were prepared in distilled sterile water, and filtered with 0.2 µm syringe filters, on the day of the experiment. *E. coli* cultures were grown in solid (1.5% agar) or liquid Luria Bertani (LB) medium, purchased from Difco, Detroit, Mi, USA, which was supplemented when necessary at the given concentrations of the tested compounds. Mueller-Hinton (Oxoid, Hampshire, UK) was employed for the determination of the TET MIC by the E-test strip (0.016–256 mg/L), purchased from AB Biodisk (VIVA Diagnostica, Huerth, Germany).

### Bacterial Strains

Wild-type *E. coli* K-12 AG100 strain (*argE3 thi-1 rpsL xyl mtl* delta (*gal-uvrB*) *supE44*) [47], was kindly offered by Hiroshi Nikaido, Department of Molecular and Cell Biology and Chemistry, University of California, Berkely, California, USA. *E. coli* ATCC 25922 was used as quality control for MIC determinations.

### Growth conditions, determination of the MIC of TET and inducing TET resistance of *E. coli* AG100

Growth conditions, preparation of inoculum and determination of the MIC by the broth macrodilution method in LB for each compound employed, and TET MIC by the E-test have been previously described [5], [47], [48]. The process by which the resistance of *E. coli* AG100 to TET was increased from 2.0 to 12.0 mg/L has been previously described [5]. Briefly, the MICs of TET for the parental AG100 strain was initially determined as 2.0 mg/L [47]. The tubes employed for the determination of susceptibility to TET that would normally be discarded after a maximum of 18 h were retained in the incubator. By the end of additional 24–48 h the tubes corresponding to concentration just above the MIC yielded evidence of growth. These cultures were tested for purity and TET susceptibility by the broth macrodilution method in LB and E-test [5], [48]. These new cultures were used to inoculate media containing increasing concentrations of TET that ranged from that from which the inoculae were prepared to higher concentrations and incubated at 37°C until evidence of full growth was present. New inoculae were prepared from the cultures that contained the highest concentration under which the strains grew. This cycle was repeated until significant increase in the resistance of the strain to TET was evident and yielded *E. coli* AG100 that were capable of growing in LB broth containing a concentration of TET as high as 10 mg/L (MICs of 12 mg/L) [5].

### Expression analyses of the membrane efflux transporter genes of the nine major *E. coli* proton-dependent efflux pump systems, outer membrane proteins and regulators, by the use of real-time reverse transcriptase-polymerase chain reaction (RT-PCR) methodology

The TET sensitive *E. coli* AG100 parent strain (MIC 2.0 mg/L) was induced to significant levels of resistance to TET by gradual step-wise exposure to the antibiotic. Transcript levels of the inner membrane efflux transporter genes of the nine major *E. coli* efflux pump systems proton pump dependent genes (*acrB, acrF, acrD, mdfA, tehA, yhiV, emrB, emrD* and *emrE*), the linker proteins AcrA and AcrE, the outer membrane channel TolC, the outer membrane proteins OmpC, OmpF, OmpA, the transcriptional regulators encoded by the multiple antibiotic resistance operon (*marRAB*) and homologs SoxS and Rob, porin transcription regulators *ompR* and *envZ*, the regulators *micF* and *ompX* , as well as the protease genes *degP, clpP, rseP* and *degS* were determined by quantitative real-time RT-PCR analyses at the end of four stages of the induction process; control culture (no TET added); the initial stage where the cells are first exposed to TET (MIC) and the cultures that grew in presence of 1.5 mg/L of TET (MIC 2 mg/L); half-way of the induction process where they grew at 4.0 mg/L of TET (MIC 6 mg/L) and at the end of the induction process where they grew at 10 mg/L of TET (MIC 12 mg/L). Gene transcript levels were normalized against the *E. coli* house-keeping gene *GAPDH* measured in the same sample. The change of the expression levels of these transporter genes, membrane proteins and regulators is presented by Figure 1 as the relative quantification of the expression level in the TET induced resistant AG100 strains relative to wild-type AG100 grown in the absence of TET at each stage of the induction process. Each result represents the average of three independent cultures grown at its respective TET resistance induction level. A ratio of 1.00 corresponds to no change of expression of the transcript levels to the parental strain.

To prevent the degradation of extracted RNA after cell lysis that might alter the expression profile of each sample at the time of harvesting, required for assuring reliable gene expression analyses, total RNA was isolated in an RNAse-free environment with the aid of the RNeasy Protect Mini Kit (Qiagen, Hilden, Germany) according to the manufacturer's instructions. The integrity, purity and concentration of the extracted RNA templates were assessed by spectrophotometry at 260 nm and agarose gel (1.5%). Purified RNA was stored in RNAse-free water in siliconised tubes and maintained at −20°C until quantification was performed.

The real-time quantification of the RNA templates by quantitative real-time one-step RT-PCR was performed in a Rotor-Gene 3000 thermocycler (Corbett Research, Sydney, Australia) strictly adhering to manufacturer recommendations of the QuantiTect SYBR Green RT-PCR Kit (Qiagen, Hilden, Germany). Briefly, each 0.2 ml standard microfuge tube contained, in a final volume of 25 µl, 12.5 µl of the 2× QuantiTect SYBR Green RT-PCR master mix, 0.25 µl of 10× QuantiTect RT mix, 900 nM of each primer and approx. 20 ng of total RNA in RNAase free water.

The primers used for real-time RT-PCR quantification of expression of each gene are described in Table 1. These were designed using Primer Express 1.5 Software (Applied Biosystems, CA, USA) based on the sequence entries in the GenBank for *E. coli* K-12 complete genome (accession number NC_000913). Primer design and PCR experimental conditions were optimised to minimise amplification of contaminating *E. coli* genomic DNA potentially present in the total RNA sample, as well as for the prevention of non-specific primer annealing. The house-keeping *GAPDH* gene (coding for D-glyceraldehyde-3-phosphate-dehydrogenase) [49] was chosen as the endogenous reference RNA for relative quantification since it revealed consistent expression levels under the experimental conditions with the primers presented in Table 1. Amplification efficiencies of the target genes and reference gene were determined through the amplification of one-step RT-PCR template dilution series and PCR conditions were optimised until comparable amplification efficiencies were obtained for identical amounts of template RNA (absolute slope values less then 0.1) from calibration curve plots according to Applied Biosystems User Bulletin #2 (Applied Biosystems, CA, USA) recommendations [50].

**Table 1:** Primers used in this study.

Efflux transporter gene and house-keeping gene	Primer sequence (5′-3′)	Length of amplicon
*marA*	CATAGCATTTTGGACTGGAT	187 bp
	TACTTTCCTTCAGCTTTTGC	
*marB*	ATAGCAGCTGCGCTTATTC	154 bp
	ACTTATCACTGCCAGTACCC	
*marR*	AGCGATCTGTTCAATGAAAT	170 bp
	TTCAGTTCAACCGGAGTAAT	
*acrA*	CTTAGCCCTAACAGGATGTG TTGAAATTACGCTTCAGGAT	189 bp
*acrB*	CGTACACAGAAAGTGCTCAA	183 bp
	CGCTTCAACTTTGTTTTCTT	
*acrD*	GATTATCTTAGCCGCTTCAA	187 bp
	CAATGGAGGCTTTAACAAAC	
*acrE*	GCCCTCCTTTATTCTGATCT	166 bp
	GGCTATACGATAAGCATTGG	
*acrF*	TAGCAATTTCCTTTGTGGTT	247 bp
	CCTTTACCCTCTTTCTCCAT	
*micF*	TCATCATTAACTTTATTTATTACCG	70 bp
	GCATCCGGTTGAAATAGG	
*soxS*	CCATTGCGATATCAAAAATC	210 bp
	ATCTTATCGCATGGATTGAC	
*rob*	GTCGTCTTTATCCTGACTCG	189 bp
	TTTGTCACCCTGGAAGATAC	
*envZ*	CGTTGAGGTCAACAAAAGTT	185 bp
	GTCGGTTCTGGATACGAATA	
*emrB*	ATTATGTATGCCGTCTGCTT	196 bp
	TTCGCGTAAAGTTAGAGAGG	
*emrD*	TGTTAAACATGGGGATTCTC	243 bp
	TCAGCATCAGCAAATAACAG	
*emrE*	GGATTGCTTATGCTATCTGG	156 bp
	GTGTGCTTCGTGACAATAAA	
*mdfA*	TTTATGCTTTCGGTATTGGT	182 bp
	GAGATTAAACAGTCCGTTGC	
*tehA*	TGCTTCATTCTGGAGTTTCT	232 bp
	TCATTCTTTGTCCTCTGCTT	
*yhiV*	GCACTCTATGAGAGCTGGTC	203 bp
	CCTTCTTTCTGCATCATCTC	
*ompF*	GAACTTCGCTGTTCAGTACC	209 bp
	CGTACTTCAGACCAGTAGCC	
*ompC*	CTTCAAAGGTGAAACTCAGG	241 bp
	GTTGTCAGAACCGTAGGTGT	
*ompA*	ACGACTGGTTAGGTCGTATG	166 bp
	ACGTTGGATTTAGTGTCTGC	
*ompX*	ACCTGAAATACCGCTATGAA	208 bp
	TCAGTGGTCTGGAATTTACC	
*ompR*	GACGTCTTCGTAGTCAGAGC	229 bp
	TTGAACTTACCGAAAGCAAT	
*degP*	TGGTAGTGAACAACGTGAAA	184 bp
	AACAGGTAGATGGTGCTGTC	
*clpP*	CAAAAGGTAAACGTTTTTGC	163 bp
	AATGATTGACCCGTATGAAG	
*rseP*	TTGTTTATTACCTGCCGTTT	190 bp
	ATTAACAGCACCAGCAGAAT	
*degS*	TTCCAGTTAGCAACCAAAAT	153 bp
	TGACACTTCATTAACCACGA	
*GAPDH*	ACTTACGAGCAGATCAAAGC	170 bp
	AGTTTCACGAAGTTGTCGTT	

Amplification of *GAPDH* and other genes of interest were then run in separate tubes using the same amount of total RNA retrieved from the same sample. Thermal cycling conditions consisted of an initial reverse transcription step at 50°C during 30 min, an initial PCR activation step at 95°C for 15 min followed by 35 cycles of denaturation (94°C, 60 s), annealing (51°C–53°C for 60 s, depending on optimised conditions for the primers used) and extension (72°C for 60 s).

The relative quantities of the mRNA of each gene of interest were determined by the use of the comparative threshold cycle (C_T_) method. Taking advantage of the fact that samples with higher initial mRNA template concentration reach the significant threshold level for real-time detection at lower PCR cycle numbers than samples containing lower initial template concentrations, it is possible to obtain a quantitative measure of the expression magnitude (ΔC_T_) of each gene of interest, normalized by the house-keeping gene (*GAPDH*) expression in each sample to correct variation in RNA content and amplification efficiencies between samples. The equation 2^−ΔΔCT^ allows the relative quantification of differences of each gene expression level between two samples, the sample of interest (the TET induced AG100 strain) and a calibrator or reference strain (the parental AG100 strain). Briefly, from three independent total mRNA extractions form *E. coli* AG100 and AG100 TET strains, grown under the described conditions, ΔC_T_ of the reference and samples for each gene tested was obtained by subtracting the C_T_ value of the *GAPDH* gene from the C_T_ value obtained for each gene. ΔΔC_T_ was calculated by subtracting the average ΔC_T_ values of the reference strain (AG100) from the corresponding TET induced ΔC_T_ for each gene tested. The relative quantifications were then calculated by the equation 2^−ΔΔCT^ as the number of fold of mRNA quantity differences relative to the calibrator or reference strain [50]. All data was collected and analysed with the aid of the Rotor-Gene 3000 real-time analysis software and the recommendations of the Applied Biosystems User Bulletin #2.

### SDS-PAGE analyses and immunoblotting

Bacteria in exponential growth phase were pelleted and solubilized as previously described [51]. Proteins were analysed on 10% SDS-polyacrylamide gel system for OmpC, OmpF, OmpA and AcrA detection and 12% SDS-PAGE gel for OmpX, [15], [51]. Gels were stained with Coomassie Brillant Blue R-250 to standardized protein samples. For western blots, proteins were electrotransfered onto nitrocellulose membranes (Schleicher & Schlull, Keene, NH, USA) in transfer buffer (20 mM Tris, 150 mM glycine, 20% isopropanol, 0.05% SDS). An initial saturating step was performed overnight at 4°C with Tris-buffered sodium (TBS: 50 mM Tris-HCl, 150 mM NaCl, pH 8) containing skimmed milk powder (10%). The nitrocellulose membranes were then incubated in TBS containing skimmed milk powder (10%) and Triton X-100 (0.2%) for 2 h at room temperature in the presence of polyclonal antibodies directed against denatured OmpC, OmpF, OmpA and OmpX, or with F4 polyclonal antibody directed against the L3 internal loop of *E. coli* porins [52]. The detection of antigen-antibody complexes was performed with alkaline phosphatase conjugated AffinitiPure goat anti-rabbit IgG antibodies (Jackson ImmunoResearch, West Grove PA, USA).
